# Supplementary material for: A piece of a puzzle—Patient and psychologist experiences of the Affect School as additional treatment in a Swedish eating disorder unit
Source: PLoS One. 2022 Jul 28;17(7):e0271902. doi: 10.1371/journal.pone.0271902 (PMC9333312; doi:10.1371/journal.pone.0271902)
Supplement: Attachment — Submitted filename: Response to Reviewers1.docx [file pone.0271902.s001.docx]

To the journal: *We wish to add “A Piece of a Puzzle” in the beginning of the title of the manuscript and hope that this will be alright.*

Journal Requirements:

2. Thank you for including your ethics statement: "The study was conducted according to the principals of the Helsinki declaration. Prior to interviews participants were provided written and oral information about the study. It was emphasised that participation was voluntary, and that participation or refusal would not affect treatment. Participants were informed that presentation of the data would be handled with confidentiality so that no statement could be traced to any single informant. The study was approved by the research ethics committee of Linköping (2017/531-31). The interviewers had no treatment relationship to the interviewees."

a) Please provide additional details regarding participant consent. In the ethics statement in the Methods and online submission information, please ensure that you have specified what type you obtained (for instance, written or verbal, and if verbal, how it was documented and witnessed). If your study included minors, state whether you obtained consent from parents or guardians. If the need for consent was waived by the ethics committee, please include this information. Once you have amended this/these statement(s) in the Methods section of the manuscript, please add the same text to the “Ethics Statement” field of the submission form (via “Edit Submission”).

## *Answer: We have added: “All participants were adults (≥18 years old).” and “Participants signed an informed consent to participation which was attached to the written information. Participants kept one copy and the other copy was saved in a safe by the researchers” to the* ***Ethical Approval and consent to participate*** *section. We have also copied the text into the Ethics Statements in the online submission information.*

3. We note that the grant information you provided in the ‘Funding Information’ and ‘Financial Disclosure’ sections do not match. When you resubmit, please ensure that you provide the correct grant numbers for the awards you received for your study in the ‘Funding Information’ section.

*Answer: Suzanne Petersson was financed by a grant from Fredrik och Ingrid Thuring’s Stiftelse (Fredrik and Ingrid Thurings’ foundation), award number: 2017-00308. The funders had no role in study design, data collection and analysis, decision to publish, or preparation of the manuscript. We cannot see where we should write to solve this problem, we cannot find the Financial Disclosure section anywhere.*

4. In your Data Availability statement, you have not specified where the minimal data set underlying the results described in your manuscript can be found. PLOS defines a study's minimal data set as the underlying data used to reach the conclusions drawn in the manuscript and any additional data required to replicate the reported study findings in their entirety. All PLOS journals require that the minimal data set be made fully available. For more information about our data policy, please see <http://journals.plos.org/plosone/s/data-availability>.

Upon re-submitting your revised manuscript, please upload your study’s minimal underlying data set as either Supporting Information files or to a stable, public repository and include the relevant URLs, DOIs, or accession numbers within your revised cover letter. For a list of acceptable repositories, please see <http://journals.plos.org/plosone/s/data-availability#loc-recommended-repositories>. Any potentially identifying patient information must be fully anonymized.

Important: If there are ethical or legal restrictions to sharing your data publicly, please explain these restrictions in detail. Please see our guidelines for more information on what we consider unacceptable restrictions to publicly sharing data: <http://journals.plos.org/plosone/s/data-availability#loc-unacceptable-data-access-restrictions>. Note that it is not acceptable for the authors to be the sole named individuals responsible for ensuring data access.

We will update your Data Availability statement to reflect the information you provide in your cover letter.

*Answer: Data Availability Statement: The raw data necessary to replicate this study consist of participant interview transcripts. Participants in this study did not consent to have their full transcripts made publicly available, and the issue of publishing interview transcripts has not been examined by the ethics review (by the Research Ethics Committee of Linköping;2017/531-31), therefore this disclosure would contravene the terms of their consent. Such restrictions have been imposed by the Region Kalmar County, who as principal for this study, is chiefly responsible for the research data collected. Data are available at the Unit for research and development at Region Kalmar län (contact* forskningssektionen@regionkalmar.se*, or phone: +46 480-426684) for researchers who meet the criteria for access to confidential data.*

5. Your ethics statement should only appear in the Methods section of your manuscript. If your ethics statement is written in any section besides the Methods, please move it to the Methods section and delete it from any other section. Please ensure that your ethics statement is included in your manuscript, as the ethics statement entered into the online submission form will not be published alongside your manuscript.

*Answer:* ***Ethical approval and consent to participate*** *is in the* ***Methods*** *section, which was earlier named “Materials and Methods.*

[Note: HTML markup is below. Please do not edit.]

To the Reviewers:

*Thank you very much for valuable and relevant comments. We have made efforts to meet your suggestions. Changes are marked with red text and commented below. We believe the manuscript has been considerably improved and hope that you now find it suitable for publication.*

Reviewers' comments:

Reviewer's Responses to Questions

**Comments to the Author**

1. Is the manuscript technically sound, and do the data support the conclusions?

The manuscript must describe a technically sound piece of scientific research with data that supports the conclusions. Experiments must have been conducted rigorously, with appropriate controls, replication, and sample sizes. The conclusions must be drawn appropriately based on the data presented.

Reviewer #1: Yes

Reviewer #2: Yes

2. Has the statistical analysis been performed appropriately and rigorously?

Reviewer #1: Yes

Reviewer #2: No

3. Have the authors made all data underlying the findings in their manuscript fully available?

The [PLOS Data policy](http://www.plosone.org/static/policies.action#sharing) requires authors to make all data underlying the findings described in their manuscript fully available without restriction, with rare exception (please refer to the Data Availability Statement in the manuscript PDF file). The data should be provided as part of the manuscript or its supporting information, or deposited to a public repository. For example, in addition to summary statistics, the data points behind means, medians and variance measures should be available. If there are restrictions on publicly sharing data—e.g. participant privacy or use of data from a third party—those must be specified.

Reviewer #1: Yes

Reviewer #2: Yes

4. Is the manuscript presented in an intelligible fashion and written in standard English?

PLOS ONE does not copyedit accepted manuscripts, so the language in submitted articles must be clear, correct, and unambiguous. Any typographical or grammatical errors should be corrected at revision, so please note any specific errors here.

Reviewer #1: Yes

Reviewer #2: Yes

5. Review Comments to the Author

Please use the space provided to explain your answers to the questions above. You may also include additional comments for the author, including concerns about dual publication, research ethics, or publication ethics. (Please upload your review as an attachment if it exceeds 20,000 characters)

Reviewer #1: The findings reported in the manuscript entitled "Patient and Psychologist Experiences of the Affect School as Additional Treatment in a Swedish Eating Disorder Unit: an Interview Study" are an important contribution to the field of eating disorders and of high relevance to clinical practice. From this reviewer's perspective, I consider that the methodology employed was suitable to achieve the objective of the present study; however, the following suggestions are made for clarity and understanding

- It is mentioned that "Parallel to the intervention both groups received treatment as usual." It would be useful to know if the type of treatment the patients had, was the same or were there differences (approach, psychological and medical treatment, psychological treatment only).

*Answer:* *They received different treatments, which we now have described in the text “which in these cases included individual psychotherapy (CBT) and in some cases also day care and/or physiotherapy”. We have also added “The patients also received different types of parallel treatments since the RCT aimed at a comparison between regular treatment, which was varying, and AS as additional treatment” to the* ***Limitations*** *section.*

- According to the manuscript, it seems that there is information on the length of time the patients have had the diagnosis of ED and its comorbidity, why not include them in the description of the sample?

*Answer: Unfortunately, we do not have reliable data on co-morbidity. But we have added the ED duration (self-referred). We also added this to the* ***Participants description****.*

- Why were the groups not formed according to diagnosis, i.e., one group of AN, another of BN, etc.?

*Answer:* *Participants were included consecutively and thus it was not possible to arrange groups with diagnose specific groups.* *This has now been added at the limitations section.*

- The discussion needs more work, as it is mainly a repetition of the results and little comparison with the existing literature.

*Answer: We have rewritten the Discussion section and hope that it will be more readable now (and with more relevant references).*

Reviewer #2: This is a study of experiences from an "Affect school" in which patients with Eating Disorders were enrolled. The study included 9 individuals who were interviewed and the responses were analysed with Thematic Analysis. Nine themes were revealed at the analysis, six from the participants, which, according to the authors, amongst others revealed that the Affect School provided an acceptance for experiencing all sorts of affects.

There are some major issues with this study:

- The study included 9 individuals which, albeit being a qualitative study, is a very small sample.

*Answer:* *Yes, the sample was 9 plus 3 AS leaders. The AS leaders were only three, thus we interviewed all of them. The number of participants in the AS group in the RCT were 21 and nine of them also agreed to participate in the interview study. In qualitative studies, you strive for a variation of parameters considered important (a broad representation) capturing different opions. The patients had different ED diagnoses and were of representative ages. The patients were also enrolled from five AS groups. Despite the small sample from different participants, the content was quite consistent which is why we considered the number of informants was sufficient.*

- the is a high risk of bias in this study where both the affect school "training", the interrelations between the enrolled subjects, and the interviewers relation to the affect school and the interviewed subjects may all have influenced the responses the subjects provided.

*Answer:* *We (the authors) are not educated in the affect school and neither of us had any relation to the interviewed AS participants (the patients). Staff was interviewed by IW,* *who only had contact with them at the time of the interview. We have now clarified this under the* ***Data collection*** *section.*

- it cannot be ruled out that the experiences were, so to speak" manipulated" by the Affect school activities.

*Answer:* *All questions were about experiences from the AS. But of course experiences and interpretations of situations always affect people's experiences, no one exists without their history and context. The possible effect of the AS was evaluated in another study.*

- some of the themes seem closely related e.g. Worries about group participation”, “Not alone anymore”, “Shared stories can also be painful”, “New insights about oneself and others”, “Relationships as a foundation for affective work”, which cast some doubt about the method for identifying themes.

*Answer:* *Time has gone (a year now) and we also think that two of the themes are related. Due to this we have re-analysed the material and combined “New insights about oneself and others” and “Not alone anymore”. We extended the name of the theme* “Relationships as a foundation for affective work” into “Relationships outside the Affect School as a foundation for affective work” *in order to clarify this theme. The reason for keeping* “Shared stories can also be painful*” is that this is a group experience which has received sparse attention in research on group interventions.*

- The conclusion that "the results showed" is very bold. This type of qualitative interview and analysis may at best provide som indication of an outcome such as "acceptance" and thereby the statement should be changed from showed to "suggest". This is also important in view of the fairly substantial bias present in this study.

*Answer:* *Yes, this is certainly correct and we have changed the word into “suggested” (as suggested).*

- Page 5, Mehods: In "Study setting" a diagnostic method is described; this should be moved to a separate section under Methods

*Answer: This is now changed according to your advice.*

- Page 5, Mehods: It is not adequate clinical praxis to diagnose different eating disorders with EDE-q and EDI alone and thus, either add information on how the diagnoses were set, or, stress that the patients were not fully diagnosed.

*Answer: No that is right. The patients were diagnosed with the Structured Eating Disorder Interview (SEDI) which was adapted to DSM-5 and not the EDI. We have added the abbreviation of the Structured Eating Disorder Interview to the text and also added that the diagnoses were set by experienced psychologists and psychotherapists, which was the case. Regarding the EDEQ, this was not a foundation for diagnosis, so we have removed this from the text.*

- Results; after each theme, the authors should indicate how many of the subjects vs AS leaders stated these (e.g. 3 of 9)

*Answer:* *Theme frequency is not to recommended in TA (Braun & Clarke, 2022)! There are many reasons not to use numbers in qualitative analysis e.g. that “more should be better”, that “numbers are better”, each data item is not directly comparable to another (it is not possible to know if those who did not mention the theme thought that the theme was not relevant).*

- Discussion; the structure is unorthodox. The authors should start by presenting and discussing the main results, thereafter other findings, thereafter limitations and finally conclude. The whole discussion needs to be restructured and rewritten.

*Answer: The sections and headings were in this order, but the Conclusion heading was missing, we apologize for this and have now completed the manuscript. We have also shortened and restructured the Discussion section in order to make it more readable.*
